# Do Cannabis Users Reduce Their THC Dosages When Using More Potent Cannabis Products? A Review

**DOI:** 10.3389/fpsyt.2021.630602

**Published:** 2021-02-18

**Authors:** Janni Leung, Daniel Stjepanović, Danielle Dawson, Wayne D. Hall

**Affiliations:** ^1^National Centre for Youth Substance Use Research, The University of Queensland, St Lucia, QLD, Australia; ^2^School of Psychology, The University of Queensland, St Lucia, QLD, Australia

**Keywords:** cannabis, marijuana, titration, THC concentration, dose

## Abstract

**Background:** Higher potency cannabis products are associated with higher risks of negative physical and psychological outcomes. The US cannabis industry has opposed any restrictions on THC levels, arguing that people titrate their THC doses when consuming higher potency products.

**Objective:** To review research on the degree to which people who use cannabis for recreational purposes can and do titrate their THC doses.

**Method:** A systematic search was conducted for studies published from 1973 to 2020. We included (1) experimental laboratory studies on dose titration of cannabis products that varied in THC content; (2) observational studies on the use of more potent products; and (3) surveys on whether cannabis users titrate when using more potent products.

**Results:** In some experiments, there were inverse associations between the THC content and the amount smoked and smoking topography, while others indicated higher doses consumed and psychological and physiological effects observed. Findings of observational studies of regular cannabis users were more equivocal. In some surveys, cannabis users reported that they use less when using more potent products, but in other surveys, persons who used more potent cannabis had more adverse effects of use.

**Discussion:** There is some evidence from experimental studies that people who use higher potency cannabis for recreational purposes can titrate their THC doses, but less evidence that regular cannabis users do in fact do so. We need much better experimental and epidemiological research to inform the design of regulatory policies to minimize harms from the use of high THC cannabis products.

## Introduction

In some states in the USA, the legalization of cannabis for adult and medical use has increased the availability and sales of cannabis products, such as extracts, that have a THC content >70% (1). The cannabis industry has resisted proposals to cap THC content by arguing that people who use high potency cannabis extracts titrate their doses (e.g., reduce their THC dosage of higher potency cannabis products to achieve the same desired psychoactive effects). They may, for example, reduce the amount smoked when using high THC products. They may also inhale smaller puffs or do so less often when using higher potency products (2). The ability to do so will depend upon users understanding the relationship between product potency and their desired effects so that they can titrate their THC dose (3).

We systematically reviewed evidence on the degree to which people who use cannabis for recreational purposes can and do reduce their THC dose when using more potent products.

## Methods

### Eligibility Criteria

We included original studies published from 1973 to the date of our search (17 June 2020) if they reported quantitative data on behavior indicative of titrating THC doses from cannabis products (*via* any route of administration) that varied in THC content (e.g., by varying or controlling the amount rolled, inhaled, or consumed).

We excluded studies of medicinal cannabis use and clinical/pharmacological studies where patients/participants were instructed to titrate their dose of cannabis consumption. Our review aimed to examine evidence on recreational cannabis use in non-supervised settings to better inform public health policy on the regulation of recreational cannabis use.

### Search Strategy

The search was conducted in PubMed and Embase with terms related to “Cannabis” AND 'Titration' in the title/abstract/related MeSH and Emtree explosion subject headings, with the “Humans” filter applied, as follows:

PubMed search: ((cannabis [tiab] OR marijuana [tiab] OR Cannabis [MeSH] OR Marijuana Use [MeSH] OR Marijuana Smoking^*^ [MeSH])) AND ((titration [tiab] OR self-titration [tiab] OR self-titrating [tiab] OR self-titra^*^ [tiab] OR titrant [tiab] OR titrat^*^ [tiab] OR auto-titration [tiab] OR autotitration [tiab])) AND (humans[Filter]).

Embase search: (((cannabis:ti,ab OR marijuana:ti,ab OR “cannabis”/exp OR “marijuana use”/exp OR “marijuana smoking^*^”) AND (titration:ti,ab OR “self-titration”:ti,ab OR “self-titrating”:ti,ab OR “self titra^*^”:ti,ab OR titrant:ti,ab OR titrat^*^:ti,ab OR “auto titration”:ti,ab OR autotitration:ti,ab)) AND “human”/de) AND (“article”/it OR “article in press”/it OR “review”/it).

The supplementary search involved the authors' collection and a snowball search of secondary references identified from all relevant records from the database search and authors' collection. Two researchers carried out the screening, study selection, and data extraction.

### Synthesis of Results

Findings from experimental and observational studies and surveys were synthesized narratively on evidence of: (1) titration behavior (e.g., amount smoked, smoking topography) and (2) evidence of effective titration, defined as adjusting consumption when using high THC products to deliver the same THC dose or to achieve the same physiological, neurobehavioral, or psychological effects obtained from using a lower dose product.

## Results

### Study Characteristics

We identified 197 records from the database search and 338 records from the supplementary search, from which, we screened 497 unique titles after exclusion of duplicates. After full-text screening (*n* = 81), we included 15 articles (Supplementary Figure 1).

Most studies were from the USA (*n* = 9), with smaller numbers from the UK (*n* = 1) (4), Canada (*n* = 2) (5, 6), and the Netherlands (*n* = 3) (2, 7, 8). Five studies were published after 2010 (2, 4, 9–11).

Experimental laboratory studies (Table 1a) recruited young volunteers who were experienced cannabis users and asked them to smoke cannabis that varied in THC concentration (e.g., (9, 10)). Observational studies (Table 1b) examined the cannabis use behavior of users (2, 4). Surveys of cannabis users (Table 1c) asked users whether they varied their patterns of use when using more potent cannabis products/assessed whether their reports of adverse effects of cannabis varied with the potency of the cannabis products that they used.

**Table 1:** Summary of experimental (a), observational (b), and survey (c) studies on titration of recreational cannabis products by potency.

**First author (year published), study location**	**Study design (sample size)**	**Sample characteristics**	**Cannabis products examined**	**Titration measure**	**Summary of findings**	**Evidence of titration behavior**	**Evidence of effective titration^†^**
**(a) Summary of experimental studies on titration of recreational cannabis products by potency**							
Cappell et al. (5), Canada	Experimental, in lab, (*N* = 12)	Experienced in cannabis use, aged 21–28, males	0.8 vs. 0.4% or 0.2% THC flower (as cigarettes)	Total time with smoke in lungs, number of puffs, mean duration of puff, mean interval between puffs, estimated weight of material consumed, finger pulse, blood pressure, and conjunctival injection.	Effective titration of intake did not occur. Number of puffs and duration for which puffs were held in lungs did not differ as a function of THC concentration. The greater the potency of the products, the more total THC participants consumed.	No	No
				Behavioral tasks: pursuit rotor, verbal memory, and raw reaction time.			
Cappell and Pliner (6), Canada	Experimental, in lab, (*N* = 60)	Frequent or infrequent cannabis use, aged 18–29 (mean = 22), males	1.45 vs. 0.73% or 0.36% THC flower (as cigarette)	Cigarette size (small and large), pulse rate, number/duration/intervals between inhalations.	There was some evidence of titration behavior with the amount of cannabis consumed increasing as potency decreased.	Yes	No
					Participants in the more potent conditions, however, self-administered more total THC, attaining the same subjective endpoint of intoxication.		
Domino et al. (12), USA	Experimental, in clinic, (*N* = 30)	Experienced in cannabis use, aged 21–33, males	2.9 vs. 0.5% (as cigarette)	Amount of cigarettes smoked/THC concentration; effects on size of palpebral fissure and pupil diameter; patellar reflex and heart rate; mood as assessed by Clyde Mood test scores.	After being asked to smoke as much as they could, participants in the higher concentrate condition had a higher increase in the amplitude of the patellar reflex and heart rate, blood pressure, pulse rate changes, and on self-reported mood.	No	No
Perez-Reyes et al. (13), USA	Experimental, in lab, (*N* = 6)	Experienced in cannabis use, aged 23–36, 50% males	2.54 vs. 1.32 vs. 1.97%	Smoking time, number of puffs, length of puff, length of hold, interval between puffs, THC plasma concentration, peak subjective high, cardiac acceleration *via* electrocardiogram (ECG).	THC cigarette consumption was dose-dependent when comparing high to low THC content, but there was no evidence of effective titration in THC plasma levels, heart rate acceleration, or reported subjective high.	Yes	No
Herning et al. (14), USA	Experimental, in lab, (*N* = 10)	Experienced in cannabis use, mean age = 29, males	3.9 vs. 1.2% (as cigarette)	Number of puffs, inter-puff interval, puff volume, puff duration, inhalation volume, inhalation duration, cumulative puff volume, cumulative inhalation volume, total smoking duration; physiological measures: heart rate, blood pressure, skin temperature, and expired CO; verbal self-report of subjective high.	The high potency cigarettes were smoked with more puffs and longer inter-puff intervals with greater inhaled volumes of air, thereby diluting the cannabis smoke. Skin temperature and intoxication rating significantly differed between low and high potency conditions.	Yes	No
Chait (15), USA	Experimental, in lab, (*N* = 10)	Experienced in cannabis use, aged 19–33 (mean = 23), 80% males	0.9 vs. 1.7 vs. 2.7% THC (as cigarette)	Amount of cigarettes smoked, cut-off time, expired carbon monoxide levels, heart rate, cigarette questionnaire (taste, harshness, draw), visual analog scales, Addiction Research Center Inventory (ARCI), mood *via* Profile of Mood States (POMS) questionnaire.	The post-smoking increase in expired air carbon monoxide levels and psychological measures did not differ between the conditions.	No	No
Heishman et al. (16), USA	Experimental, in lab, (*N* = 12)	Experienced in cannabis use, aged 23–43 (mean age = 31), males	2.7 vs. 1.3 vs. 0%	Heart rate, smoking topography (inter-puff interval, puff duration, puff volume, maximum flow rate/puff, average flow rate/puff); subjective report of drug effects; a cognitive battery measuring working memory, attention, and motor ability (digit-symbol substation task).	Participants in the high dose condition took smaller puffs, lesser inhalation volumes and shorter puff duration, but did not differ in other smoking topography measures.	Yes	No
					There was no effect on attention—digit span/symbol substation tasks results did not show a dose-response effect. However, subjective reports of dose-related effects of cannabis were obtained.		
Matthias et al. (17), USA	Quasi-experimental, in lab, (*N* = 10)	Experienced in cannabis use, mean age = 23, males	3.95 vs. 1.77 vs. 0%	COHb saturation, self-report subjective level of intoxication, volume and number of puffs and inter-puff intervals, inhaled volume, breath-holding time, respiratory THC retention, heart rate.	Participants in the stronger dose condition showed reduced intake of smoke and tar yield. THC retention and heart rate were increased in the higher THC concentrations.	Yes	No
Hartman et al. (10), USA	Experimental, in lab, (*N* = 19)	Used in past 3 months, at most three times/ week, aged 21–37, 72% males	Placebo (0.008%), low (2.9%), vs. high (6.7%) THC; ground bulk cannabis vaporized *ad-libitum* for 10 min	Blood and plasma cannabinoid analysis.	Of participants that completed all experimental sessions, 10 showed self-titration as indexed by maximum blood THC concentration (μg/L).	–	Mixed
				No behavioral measures of titration presented.	Low concentration cannabis sessions produced consistent max concentration and AUC values in participants, whereas high dose products did not.		
Hartman et al. (9), USA,	Experimental, in lab, (*N* = 19)	Used in past 3 months, at most three times/ week, aged 21–37, 72% males	Placebo (0.008%), low (2.9%), or high (6.7%) THC; ground bulk cannabis vaporized vs. libitum for 10 min	Oral fluid THC concentration.	Max THC concentrations in oral fluid were higher in active (low and high) dose cannabis conditions than placebo. No difference in oral fluid THC were detectable between low and high dose overall or at any timepoint post-dose. Given that differences in blood and plasma were detected in some participants, this suggests a failure of oral fluid THC sensitivity.	–	Mixed
				Blood and plasma cannabinoid (also reported in Hartman et al. (10)).			
Bidwell et al. (11), USA	Experimental (between-subjects), sample recruited *via* social media and mailed flier adverts, (*N* = 121)	Experienced flower or concentrate use, mean age = 28, 55–64% males	Concentrates (70 vs. 90%) or flowers (16 vs. 24%)	Plasma cannabinoids; subjective drug intoxication; mood *via* modified POMS questionnaire; neurobehavioral tasks testing memory, inhibitory control, eyes open, and closed balance.	THC exposure was significantly higher in the concentrates conditions. Neuro-behavioral outcomes did not differ by potency.	–	Mixed
**(b) Summary of naturalistic observational studies on titration of recreational cannabis products by potency**							
Freeman et al. (4), United Kingdom	Naturalistic observational, recruited by word-of-mouth and snowball sample, (*N* = 247)	Used daily, mean age = 20, 74% males	Own cannabis products varying in potency (1–10) and type (skunk, resin, or herbal); samples analyzed for THC concentrations	Consumption behavior observed from participants smoking their own cannabis in front of the researcher. Self-reported subjective intoxication. Verbal IQ assessed using Wechsler Test of Adult Reading (WTAR).	There was a negative association between THC concentration and amount of cannabis used, but non-daily users were poor in potency estimation. Cannabis product type influenced THC and CBD concentrations. User-estimated potency, in turn, differed as a function of product type, and therefore potency. Amount of product consumed was not influenced by product type/potency. Subjective intoxication did not differ.	Yes	Incomplete
van der Pol et al. (2), Netherlands	Naturalistic observational, “coffee-shops” and chain referral sample, (N=98)	Experienced in cannabis use, aged 19–32 (mean = 24), 75% males	Own products varying in THC concentration, and comparisons of 15.72 vs. 3.64%	Smoking topography measured using a portable device [puff volume, duration, inter-puff interval, average velocity (ml/second), peak flow (ml/second), time to peak puff velocity (ml)].	Higher THC concentration was associated with lower inhalation volume and pace, but not with other topography measures, and positively associated with amount used. The sub-group who used the highest THC product (15.72%) inhaled less than users of average products (3.64%), but the inhalation only halved when the THC concentration was four times higher.	Mixed	Incomplete
**(c) Summary of survyes of cannabis users on titration of recreational cannabis products by potency**							
Reinarman (8), USA & Netherlands	Household survey, (San Francisco *N* = 266; Amsterdam *N* = 216)	Experienced in cannabis use, mean age = 34–37, 53–59% males	“Stronger cannabis”	One self-report item: “When using stronger cannabis, do you use…” Less, Same, or More?	Seventy percent of participants self-reported that they use less when using stronger cannabis	Yes	–
Korf et al. (7), Netherlands	“Coffee-shops” field interviews, (*N* = 388)	Smoked cannabis in last 30 days, mean age = 28, 79% males	Own products with dosage assessed using a prompt card showing 0.05, 0.10, 0.20, 0.30 g of cannabis/hash	Self-report of (a) consumption characteristics measured using validated tools, and (b) self-adjustment behaviors in the hypothetical situations that they were smoking more potent products.	Three broad types of cannabis users were identified with mixed results. The type who preferred milder cannabis reported compensating by inhaling less deeply and smoking less. However, the youngest group who consumed the highest monthly dose reported inhaling more deeply, and the oldest group did not report adjustments to intake.	Mixed	–

*–, not reported; –, not assessed; AUC, area under the concentration-time curve; CBD, cannabidiol; CO, carbon monoxide; COHb, Carboxyhemoglobin, carbon monoxide that formed when inhaled; THC, tetrahydrocannabinol; POMS, Profile of Mood States*.

† *Effective titration was defined as adjustments in consumption behavior when using high THC products that resulted in no increase in THC exposure or no differences in neurobehavioral effects*.

### Narrative Review

#### Experimental Laboratory Studies

There was mixed evidence of titration in experimental studies that were conducted in the 70–90's (5, 6, 12–17). These studies used various methods to measure dose titration (e.g., measuring the total amount of THC that was self-administered and assessing the physiological, and psychological effects of the cannabis consumed).

Some of these studies reported differences in smoking topography, such as taking smaller puffs, smaller inhalation volumes, shorter puff duration, longer inter-puff intervals, when using more potent cannabis products (6, 13, 16). Other studies did not (5, 12, 15). The participants in the higher dose conditions in all studies consumed more THC and reported more psychoactive effects, regardless of adjustments in their smoking behavior.

More recent studies have found some evidence of titration. Hartman et al. conducted an experimental study that evaluated the cannabinoid levels in blood and plasma after the use of vaporized cannabis that varied THC content, with and without alcohol consumption, while allowing *ad-libitum* consumption (9, 10). They recruited 32 participants who had used cannabis in the past 3 months no more than three times a week. Nineteen (59%) completed all the sessions and provided data on cannabinoid levels in blood and plasma concentrations (10) and oral fluid (9). Participants inhaled vaporized cannabis (ground cannabis obtained through NIDA) *ad-libitum* for 10 min. The THC levels were 0.008% in the placebo, 2.9% in the low and 6.7% in the high concentration cannabis conditions. Participants consumed the three cannabis products with and without a concurrent low-dose alcoholic beverage, across six testing sessions.

In the analyses of blood and plasma THC concentrations (10) 10 of the 19 participants showed evidence of dose titration as indexed by maximum blood THC concentration (μg/L). Specifically, four participants had THC concentrations for the low and high concentration conditions were within 20% of each other, and six participants had greater THC concentrations in the low than the high cannabis condition. Sessions using low concentration cannabis produced consistent THC blood max concentration and AUC values whereas higher dose products did not. This suggests that users attempted to titrate their dose. Data were not presented separately for alcohol and no alcohol conditions, but there were no significant interactions between cannabis dose and alcohol consumption in their effects on THC concentration or AUC.

Bidwell et al. (11) reported a between-subjects experimental study in cannabis users who predominantly used flower/concentrates. They measured blood levels of cannabinoids and the active THC metabolite 11-hydroxyΔ^9^-THC (11-OH-THC) and assessed subjective intoxication and mood, and performance on memory, inhibitory control, and balance. Participants were randomly assigned to smoke cannabis products of their preferred type that were standardized to contain either low (flower: 16%; concentrate: 70%) or high (flower 24%, concentrate 90%) THC concentrations.

Blood THC and THC metabolite levels differed between the two forms of cannabis, with concentrates producing higher blood levels than flower (11). There was no significant difference in levels between the two potency levels for cannabis concentrate (70 vs. 90% THC). For cannabis flower, the difference in blood levels approached the pre-specified significance threshold of *p* < 0.01 for blood THC (*p* = 0.01) and 11-OH-THC (*p* = 0.02). Although this effect was not nominally significant, it suggested that participants who predominantly used flower experienced more difficulty adjusting their THC intake.

Concentrate users achieved more than double the mean blood THC level of flower users (11). Despite this difference, self-reported measures of intoxication did not differ between users of the two products. The reason for this discrepancy is unclear. Possible explanations include increased tolerance to THC in concentrate users, a saturation of the cannabinoid receptors so that additional THC intake no longer produced an effect, or differences in user characteristics that affect metabolism/sensitivity to THC. Potency did not significantly affect any of the neurobehavioral measures.

#### Observational Studies

Observational studies of cannabis users' behavior when using cannabis that varied in potency have shown mixed evidence of titration (2, 4).

Freeman et al. (4) reported an observational study in the UK in which participants used their own cannabis that chemical analyses had established varied in potency and type (skunk, resin, and herbal). Participants were asked to roll a joint and smoke it normally while the researcher recorded their self-reported subjective intoxication and assessed their verbal IQ using the Wechsler Test of Adult Reading.

The study found a negative relationship between THC concentration of the cannabis and the amount of cannabis added to their joints. This relationship was not influenced by the users' frequency of use. The THC levels of the cannabis products were positively correlated with participants' estimation of their potency but the correlation was low. The amount of cannabis consumed was not influenced by product type/potency and participants did not differ in their subjective levels of intoxication.

A similar study in the Netherlands by van der Pol et al. found mixed evidence on whether experienced cannabis users could successfully titrate their THC doses (2). This was a naturalistic, observational study of young experienced cannabis users recruited through “coffee-shops” and chain referrals. The participants used their own cannabis products that varied in THC concentration. Smoking topography was measured using a portable device to assess puff volume, duration, inter-puff interval, average velocity (ml/s), peak flow (ml/s), and time to peak puff velocity (ml).

van der Pol et al. (2) found a positive association between cannabis THC concentration and the amount of cannabis consumed (i.e., participants who used more potent cannabis used larger amounts in their regular joints). There was, however, a negative association between THC concentration of joints and total inhaled smoke volume. This indicated that users inhaled less cannabis smoke when using cannabis with higher THC concentrations. Despite this, they consumed larger amounts when using high potency cannabis. This suggests that their attempt to titrate their doses was only partially successful as measured by THC in blood plasma.

#### Surveys of Cannabis Users' Behavior

A survey comparing patterns of cannabis use in San Francisco and Amsterdam is often cited as evidence for titration (8). In this study cannabis users were asked: “When using stronger cannabis, do you use less, same, or more?” One-third of respondents reported that they used the same amount, and two-thirds reported that they used less. Those who reported smoking less of “stronger cannabis” said that they preferred to achieve the same effect by using less cannabis. This study can be considered as hypothesis-generating, because it did not employ puff topography or measure THC.

Korf et al. (7) conducted a survey of Netherlands “coffee-shop” patrons who used cannabis and hash products that varied in potency. They collected data on self-reported behavior when smoking more potent cannabis products and identified three groups of users. The first group that varied in age and sex and preferred to use milder cannabis reported inhaling less deeply and smoking smaller amounts of higher potency cannabis. The second was a younger group with more symptoms of cannabis dependence who reported that they inhaled more potent products more deeply. The third comprised older predominantly males with long cannabis careers who lived and smoked alone. They did not report any adjustments in smoking behavior when they used more potent cannabis.

## Discussion

This review found mixed evidence on how successful cannabis users were in adjusting their dose of more potent cannabis to achieve the same delivery of THC or the same desired psychoactive effects. Older experimental studies found little evidence for titration but often used cannabis with much lower THC levels that differed minimally between conditions. More recent experimental studies of *ad-libitum* cannabis provided some evidence of titration by finding reductions in the amount of THC in blood and plasma when products of different potency were used. An experimental study of controlled cannabis vaporization found similar THC concentrations in blood in the low and high dose THC conditions in some, but not all, participants. This provides some support that some cannabis users titrate their THC dose during *ad-libitum* consumption (9, 10).

Observational studies found weak evidence that cannabis smokers reduced their THC doses when using cannabis products with higher levels of THC. In surveys, there were self-reported changes in cannabis use but no assessments were made of whether these produced differences in the THC dose consumed or in its physiological or psychological effects.

The question of most relevance to cannabis policy is whether the users of higher THC products do, in fact, titrate their doses. Epidemiological surveys of adverse effects reported by cannabis users suggest that users of more potent cannabis products incompletely adjust their THC doses. In these surveys, consumers of higher THC cannabis products report more negative consequences than users of less potent products (18, 19). A UK cohort study showed that users of high potency cannabis had higher risks of generalized anxiety and cannabis use disorders (20).

There are supportive trends in ecological data. In the USA emergency, hospital, and poisoning center presentations related to cannabis have increased along with the increased use of high THC cannabis products after cannabis legalization (21). In the Netherlands, there was an increase in the number of persons seeking help to quit cannabis as the average THC content of cannabis sold in coffee shops increased and a later fall in numbers when THC content declined (3).

### Limitations of the Evidence

This review was severely limited by the dearth of rigorous studies on whether people who use cannabis can effectively titrate their doses of higher potency cannabis. The recent rapid increase in THC potency in cannabis products on the market makes it difficult to compare the findings of early studies that used very low THC cannabis products by comparison with cannabis products now consumed.

There may also have been changes over time in the characteristics of people who use cannabis and in their frequency of use. Tolerance develops with the frequency of cannabis intake so cannabis effects will differ between the occasional users often studied in laboratories and the daily cannabis users who account for most of the cannabis consumed (22).

Routes of administration have also changed over time. Although we did not restrict our search to studies of any specific route of administrations, all the studies we included were of inhaled cannabis products. Methods and ease of titration between different routes of cannabis administration may vary. Vaporization and smoking provide similar cannabinoid delivery (23), but the subjective effects of edible products have a longer time course and users may be at risk of consuming more than intended if they had not waited for them to take effect before deciding to consume more. There are doubts about how well users can titrate their THC doses of oral cannabis products, given that individuals may not know how long they need to wait to assess whether they have reached their desired level of intoxication. Future studies are needed on self-titration of cannabis use by new and emerging administration methods.

Some early laboratory studies of cannabis consumption assessed the relationship between blood concentrations of THC and the effects of cannabis (24). However, many surveys have only assessed titration by self-report rather than measuring the THC content of cannabis or the level of users' intoxication. Self-reported titration can be subject to selective reporting, memory effects and bias and hence provides weak evidence for the titration.

Smoking topography was measured in some studies, with some authors arguing that it is difficult to assess titration without these measures (16). The use of behavioral endpoints as measures is problematic because frequent users have higher tolerance. Objective measures of cannabis potency and THC exposure, such as assessing THC concentration in blood and plasma in laboratory settings, are required in future research on cannabis dose titration.

This review was restricted to papers written in English. The predominance of studies from North America may limit the generalisability of these results. The marketing of high THC content products in the USA may have global impacts as online markets are increasingly popular and merchants accessible through online crypto-markets in the USA are prepared to ship cannabis products worldwide (25).

Our review excluded studies of cannabis when used to alleviate symptoms of chronic medical or mental health conditions. Future research is needed that monitors the prevalence of medical use and assesses the extent to which medicinal cannabis users titrate their doses.

There is an urgent need for larger and better controlled experimental and observational studies of the extent to which cannabis users can and do titrate their THC doses when using more potent cannabis products, such as, cannabis extracts and high potency cannabis flower. This research is needed to inform policymakers on how to reduce harms from the use of high potency cannabis products. It may indicate the need for caps on the potency of cannabis products or higher taxes on more potent cannabis products to discourage their heavy use (26). It is also needed to inform the labeling of THC doses in legal cannabis products that may include standardized THC doses analogous to standard units of alcohol (27).

## Author Contributions

WH and JL: design and conception. JL, DS, and DD: acquisition and analysis of data. JL and DD: first draft. All authors: interpretation of data, subsequent drafts, revision for important intellectual content, final approval, and agreement to be accountable for the work.

## Conflict of Interest

The authors declare that the research was conducted in the absence of any commercial or financial relationships that could be construed as a potential conflict of interest.
