# Supplementary figures and images for: Do Cannabis Users Reduce Their THC Dosages When Using More Potent Cannabis Products? A Review
Source: Front Psychiatry. 2021 Feb 18;12:630602. doi: 10.3389/fpsyt.2021.630602 (PMC7930233; doi:10.3389/fpsyt.2021.630602)

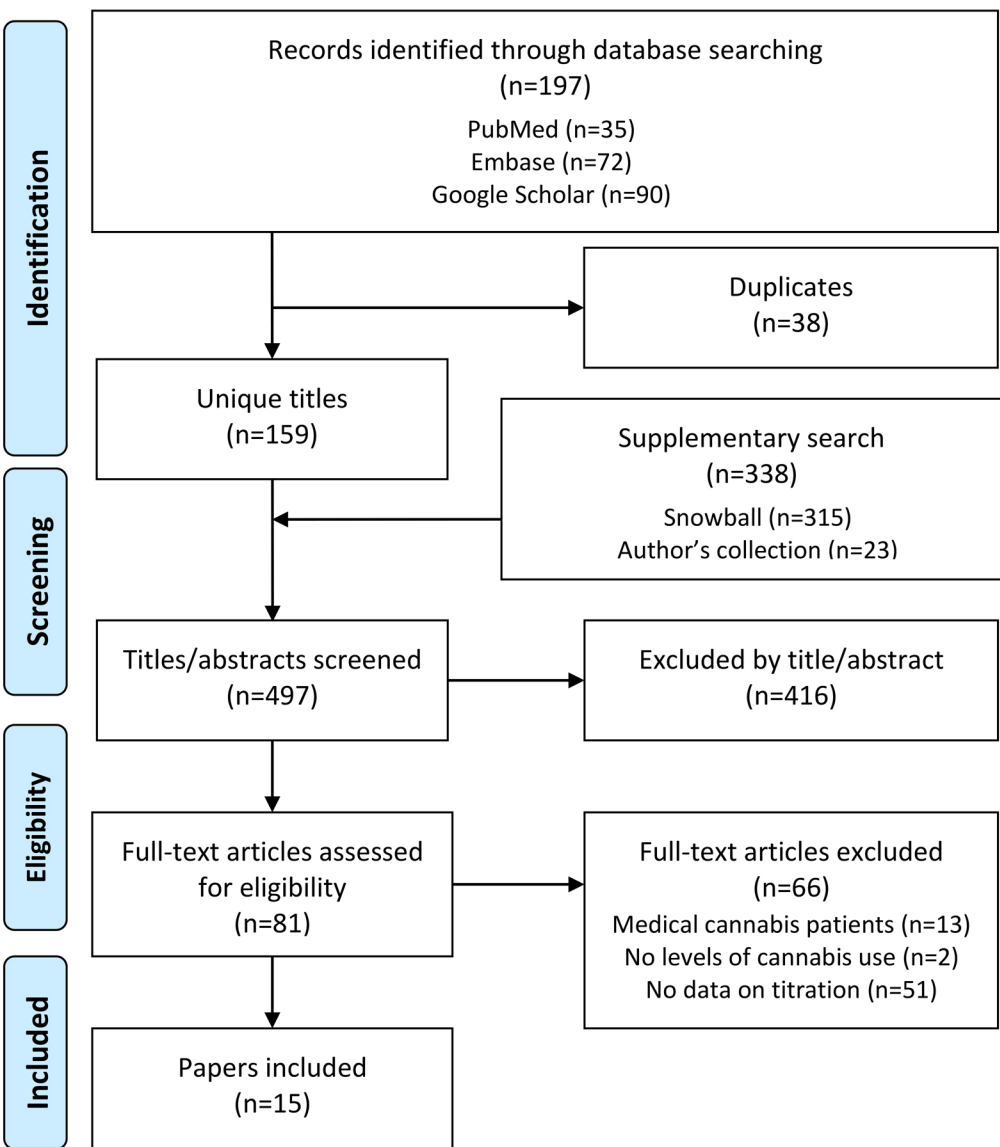

Figure 1. PRISMA flowchart

Supplement: Supplementary file 1 [file Image_1.PDF]
